# Remarks on the Arteries

**Published:** 1816-08

**Authors:** George Kerr


*For the London Medical and Physical Journal.*

### *Remarks on the Arteries;* by George Kerr, Esq.

THE account given in your last Number of Dr. Parry's Experiments concerning the Arterial Pulse, is highly interesting, particularly those showing the manner in which new arterial shoots connect the upper and under part of an artery, when the cavity is obliterated by ligature.

A very material point to be ascertained is, in what time these new vessels are formed, and, whether those which Dr. Parry found incapable of receiving coarse injection after twelve twelve months would have been at all visible during the first month after the ligature was applied. In the second volume of the Transactions of the Medical and Chirurgical Society, Mr. Astley Cooper describes an experiment in which the descending aorta of a dog was tied: the animal seemed to feel no extraordinary inconvenience, and, when killed, about thirteen months afterwards, four or five small arteries were found connecting the part of the artery above the ligature with that immediately below. I would submit the question to you and your ingenious correspondents,—"In what manner could the circulation of the blood go on till these new vessels were formed?" According to Harvey's doctrine, the motion of both sides of the heart is occasioned by the distension by blood, and the whole blood is said to pass through the system many times in an hour. In the case given by Mr. Cooper, the heart continued its operations, and the *cava ascendens* must have been deprived of its contents *(exantlato sanguine)* within a very few minutes; a fatal congestion about the heart, lungs, head, and fore-limbs, being the consequence. To me it seems, that the dog could not possibly wait ten minutes for the production of the new vessels, if we suppose that the right side of the heart is every pulsation exhausting the blood of the veins; for thus the whole contents of the blood-vessels of nearly one-half the body would be rapidly accumulated on the vital parts. The venous blood has been said to acquire an *impetus* from the extreme arteries; but, as these arteries escape the examination of our senses, having no sensible pulsation, and no remarkable swelling of the foot or leg taking place after the saphena has been tied, and the artery of the limb left free, I cannot but regard this supposed *impetus* as a mere gratuitous assumption. My difficulty therefore remains, how I can account for the existence of the dog during the time immediately succeeding the operation, when no new connecting vessels can be conceived to exist, and the heart goes on performing its usual operations.

*Aberdeen;*
*June* 18, 1816.

P. S. Colchicum is no new remedy for gout; it was in use among the earlier Greek physicians, and is mentioned as the principal remedy by Pepagomenus, who wrote a Treatise on Gout, at the request of the Emperor Michael Palæologus, in the thirteenth century.

